# An All-Fiber-Optic Combined System of Noncontact Photoacoustic Tomography and Optical Coherence Tomography

**DOI:** 10.3390/s16050734

**Published:** 2016-05-20

**Authors:** Jonghyun Eom, Jun Geun Shin, Soongho Park, Sunghwan Rim, Byeong Ha Lee

**Affiliations:** 1 Department of Medical System Engineering, Institute of Integrated Technology, Gwangju Institute of Science and Technology, 123 Cheomdan-gwagiro, Buk-gu, Gwangju 61005, Korea; eomjonghyun@gist.ac.kr; 2 School of Information and Communications, Gwangju Institute of Science and Technology, 123 Cheomdan-gwagiro, Buk-gu, Gwangju 61005, Korea; jgshine@gist.ac.kr (J.G.S.); shpark88@gist.ac.kr (S.P.); rim@gist.ac.kr (S.R.)

**Keywords:** fiber-optic imaging, interferometry, multimodal imaging, noncontact measurement, optical coherence tomography, photoacoustic imaging

## Abstract

We propose an all-fiber-based dual-modal imaging system that combines noncontact photoacoustic tomography (PAT) and optical coherence tomography (OCT). The PAT remotely measures photoacoustic (PA) signals with a 1550-nm laser on the surface of a sample by utilizing a fiber interferometer as an ultrasound detector. The fiber-based OCT, employing a swept-source laser centered at 1310 nm, shares the sample arm of the PAT system. The fiber-optic probe for the combined system was homemade with a lensed single-mode fiber (SMF) and a large-core multimode fiber (MMF). The compact and robust common probe is capable of obtaining both the PA and the OCT signals at the same position without any physical contact. Additionally, the MMF of the probe delivers the short pulses of a Nd:YAG laser to efficiently excite the PA signals. We experimentally demonstrate the feasibility of the proposed dual-modal system with a phantom made of a fishing line and a black polyethylene terephthalate fiber in a tissue mimicking solution. The all-fiber-optic system, capable of providing complementary information about absorption and scattering, has a promising potential in minimally invasive and endoscopic imaging.

## 1. Introduction

Photoacoustic tomography (PAT) is a powerful noninvasive and nonionizing biomedical imaging modality that has attracted great attention in the last decade [1,2,3,4]. PAT is based on the optical absorption and detects laser-induced ultrasound waves via the photoacoustic (PA) effect [5]. When a short pulse from a laser illuminates a biological sample, a portion of the laser energy is absorbed in chromophores such as hemoglobin, melanin, *etc.* within the sample. The absorbed optical energy is converted into a thermal energy, leading to thermal expansion and generation of broadband ultrasonic waves (referred to PA waves), sequentially. The generated PA waves are detected by a conventional ultrasound transducer or an ultrasound transducer array. The PA images are then reconstructed by using acoustic back propagation algorithms [1,2,3,4]. Since the PA signal is strongly associated with the optical absorption properties of the sample, PAT can provide optically absorbed structural (*i.e.*, vascular structure and internal organs), functional (blood oxygen concentration, blood flow, and hemodynamic change), and molecular (tumor and cancer cell) images of various biological tissues [1,2,3,4]. Especially, PAT is capable of enhanced depth imaging compared with the traditional optical imaging modalities using only the ballistic scattering regime. However, PAT is less sensitive to the low-absorptive constituents in tissue, hence having difficulty in visualizing the tissue morphology.

Optical coherence tomography (OCT) is a high-resolution and noncontact imaging system that uses low-coherence interferometry. OCT is capable of having 2D and 3D tomograms of the internal microstructures of various biological samples, based on scattering and reflecting [6,7]. The OCT signal is obtained from the interference between the beam back-reflected from the reference arm and the light back-scattered from the internal layers of the sample, which depends on intrinsic refractive index gradients. However, the OCT image is restricted in presenting the optical absorption property of the sample and in-depth imaging up to 2~5 mm due to the short mean free path of biological tissues. Therefore, the combination of PAT and OCT can potentially provide valuable complementary information of structural and functional features of biological tissues.

Recently, the dual modality of PAT and OCT has been reported [8,9,10,11]. In practice, however, the conventional PAT system that uses an ultrasound transducer for detecting PA signals in water has a number of challenges on system configurations to be combined with an OCT system, because it employs a different method for signal acquisition. In previously reported PA-OCT systems, PA excitation and OCT beams were incident on one side of a sample and the detection was made with an ultrasound transducer at the other side [8,9], or both beams and the ultrasonic transducer were placed side by side—not in-line [10]. This detection arrangement is only suitable for thin samples, which are usually used in PA microscopy imaging. Moreover, this different detection method presents an obstacle to combining PAT and OCT signals with one detecting probe or to making in-line common detection to provide a co-registered PAT and OCT image of the same location. In addition, using two different detectors inevitably leads to complex alignments and huge system configurations including the usage of water containers and bulky optic probes [8,9,10,11]. Consequently, the existing combined PAT-OCT system has big challenges for application in minimally invasive imaging and endoscopy.

To overcome these limitations of the PAT-OCT systems, several studies have been demonstrated using optical interferometric methods [12,13,14,15,16]. A thin-film Fabry-Perot interferometer [12,13], a low-coherence Michelson interferometer [14,15], and a fiber-type Mach-Zender interferometer [16] have been utilized for PA signal detection instead of the conventional ultrasound transducer and for sharing the sample arm with OCT. However, these techniques are still impertinent for minimally invasive or endoscopic imaging due to bulky optics configuration [12,13,14] and a contact-based detection scheme [12,13]. Fiber-based systems [15,16] have also been proposed for the noncontact PAT and OCT. However, they are still composed of bulky optics in the probe part, and a bulk-type pulsed laser delivered through free space is used for PA excitation. Thus, these systems are also unsuitable for minimally invasive and endoscopic imaging.

In this paper, we present an all-optical-fiber-based dual-modal PAT-OCT imaging system employing a compact common probe, which shares the sample arm of both a noncontact PAT sub-system and a swept-source OCT sub-system. The fiber-based noncontact PAT and the miniaturized probe can readily overcome the aforementioned limitations of the combined system configuration. As both PAT and OCT probe beams are guided into the same fiber-optics probe, it is possible to acquire the PAT and OCT signals at the same detection position. In addition, the fiber-optic design of the probe is compact, simple, flexible, cost-effective, robust, and stable. Further, it is free from electromagnetic interference. Thus, it can be beneficial for commercialization and clinical applications in fields requiring minimally invasive or endoscopic imaging. To the best of our knowledge, this fiber-based probe is the mostly miniaturized among the existing combined systems. To evaluate the performance of the proposed dual-modal PAT-OCT imaging system, we conducted a tissue-mimicking phantom experiment, which was designed to provide both the absorption and the scattering contrast tomographic images.

## 2. Materials and Methods

**Figure 1:**
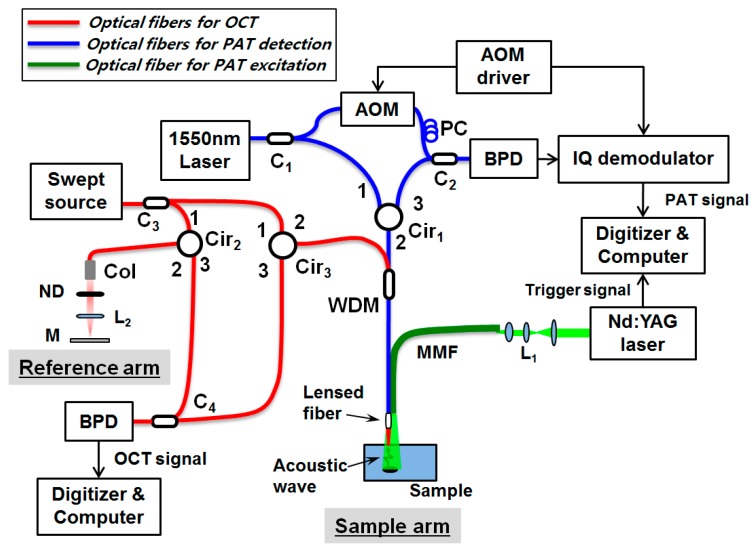
Schematic of the all-fiber-based dual modality of photoacoustic and optical coherence tomography with a miniature common probe. AOM: acousto-optic modulator; PC: polarization controller; BPD: balanced photodetector; C_1_–C_4_: fiber coupler; Cir_1_–Cir_3_: fiber circulator; Col: collimator; ND: neutral density filter; L_1, 2_: lens; M: mirror; WDM: wavelength division multiplexer; MMF: multimode fiber.

The proposed all-fiber-based combined PAT-OCT system is depicted in Figure 1. The system configuration is based on two fiber-based Mach-Zender interferometers; one for the noncontact PAT system (blue lines) and the other for the OCT system (red lines). The common fiber-optic probe, made up of a lensed single-mode fiber (SMF) and a large-core multimode fiber (MMF), is used for the common sample arm of two systems.

### 2.1. All-Fiber Nonconact PAT System

The noncontact PAT system using the fiber-interferometric detection scheme has been previously reported [17,18]. The surface displacement of a sample, induced by propagating PA waves, was measured by a heterodyne interferometer. However, the PA excitation laser beam was delivered, separately from the detection beam, through free space by using bulk optics. For getting all-fiber configuration, in this study, we have modified the previous scheme as depicted with blue and green lines in Figure 1. In brief, a Q-switched Nd:YAG laser (Brilliant ultra 50, Quantel, Paris, France) with a pulse width of 8 ns, center wavelength of 532 nm, maximum output energy of 50 mJ per pulse, and repetition rate of 20 Hz is used for PA excitation. It is coupled into a MMF (wf600/660, CeramOptec, Bonn, Germany) and guided to illuminate a sample in a narrow imaging area. The generated PA waves are detected by the fiber-based heterodyne Mach-Zender interferometer [17,18] at the sample surface, which allows for the noncontact PA measurement. For the heterodyne interferometer, a single frequency laser (SFL-1550, Thorlabs, Newton, NY, USA), with a wavelength of 1550 nm and maximum output power of 30 mW, is used. The output light from the source is coupled into a SMF and splits into reference and sample arms by a 1 × 2 fiber coupler (C_1_). In the reference arm, the beam is frequency modulated with an acousto-optic modulator (AOM; Fiber-Q FCAOM, Gooch & Housego, Ilminster, UK) and directed into the second coupler (C_2_) after passing through a polarization controller (FPC031, Thorlabs). The sample beam goes through a circulator and a wavelength division multiplexer (WDM; WDM-1310/1550, Fiberpia. Co. Ltd., Daejeon, Korea) and is then focused onto the sample via the self-built lensed fiber.

The WDM allows for the integration and separation of two light sources; the OCT light and the PA signal interrogating light. The beam reflected from the sample surface is recoupled into the same microsized fiber probe. Thereafter, the reference and the sample beams interfere with each other at the fiber coupler (C_2_), and are then detected by a balanced photodetector (PDB430C, Thorlabs). Before digitizing, an in-phase and quadrature (IQ) demodulator is used for demodulation of the detected interference signal, which carries the phase information of surface displacement. A high-speed digitizer (NI-5142, National Instruments, Austin, TX, USA, 100 MS/s) samples the demodulated signals, and the surface displacement induced by the PA wave is calculated from the recorded signals. Finally, the PA signals are estimated by taking a time derivative of the surface displacement [19]. By scanning the fiber-optic probe with a motorized linear translation stage (PM-500, Newport Co., Irvine, CA, USA), the PA signals are acquired at different locations. The PA image is obtained by the Fourier transform-based reconstruction algorithm with the measured PA signals [20]. The spatial resolution of the noncontact PAT has been previously reported [18]; briefly, the lateral and axial resolutions of PAT system at a depth of 2.5 mm were 100 μm and 30 μm, respectively. With an increase in the imaging depth, the lateral resolution became poor, whereas the axial resolution remained almost constant.

To achieve the noncontact measurement of PA waves, the heterodyne optical interferometer was utilized. It is generally known that the heterodyne optical interferometer is strong to interference artifacts coming from environmental variations, spurious noise, layers in the sample *etc.*, because the detection is made at the high frequency region, where the noise is low, with the help of a high frequency modulation [21].

### 2.2. The OCT System

Swept-source OCT is consisted of fiber optics as well, which is described with the red lines in Figure 1. A wavelength sweeping laser (HSL-2000, Santec, Komaki, Japan) with a center wavelength of 1310 nm, a wavelength bandwidth of 110 nm, a scan speed of 20 kHz, and an average power of 10 mW was used as the light source. The beam from the swept-source was coupled into a SMF and divided into the reference and sample arms of the Mach-Zender interferometer using a 1 × 2 coupler (C_3_). The sample arm of OCT shared the lensed SMF with the sample arm of the PAT system through the WDM. In the reference arm, a collimator, a ND filter, a lens, and a mirror were used to match the optical path lengths of two arms. The light back-scattered from a sample was collected by the lensed fiber and recombined with the light reflected from the reference arm at a 1 × 2 coupler (C_4_). Then, the spectral interferogram formed with the two beams was detected by a dual balanced photodetector (Model 1817, New Focus Inc., Irvine, CA, USA) and sampled by a digitizer (PX144000A, Signatec Inc., Newport Beach, CA, USA) with a sampling rate of 400 mega samples/s and 4096 points in each A-scan. The data interpolation for resampling to k-space was performed to each A-line datum prior to processing of FFT. OCT signal processing was conducted by an on-board field programmable gate array (FPGA) of the digitizer [22] to reduce the processing load of the host computer. The measured axial resolution was 8.8 μm in air.

### 2.3. Fiber-Optic-Based Sample Probe

**Figure 2:**
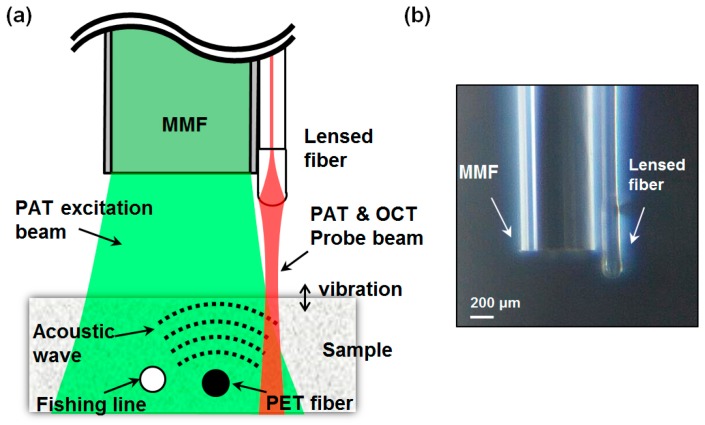
(**a**) Schematic and (**b**) photograph of the all-fiber microsized probe. It is composed of a lensed single-mode fiber (SMF) for optical coherence tomography (OCT) and photoacoustic tomography (PAT) interrogation, and a multimode fiber (MMF) for PA excitation.

Figure 2 shows the schematic of the proposed all-fiber-optic sample probe and the photograph of an implemented one, which is consisted of the self-built lensed fiber and the large-core MMF. The lensed fiber was fabricated by splicing a SMF with a short piece of a coreless silica fiber (CSF) and then by forming a lens on the other end of the CSF with a fusion splicer (S183PM, Furukawa FITEL Co., Phra Nakhon Si Ayutthaya, Thailand) [23]. The SMF had core/cladding diameters of 9/125 μm, and the CSF had a diameter of 180 μm. The length of the CSF piece was 600 μm. Probing beams of PAT and OCT, integrated by the WDM, were expanded in the CSF and then focused onto a sample by the micro-size lens at the fiber tip. The PAT probing beam reflected at the sample surface and the OCT probing beam back-scattered or back-reflected at inside the sample were collected by the same fiber lens and guided through the core of the SMF probe.

The Q-switched Nd:YAG laser beam was coupled into the large-core MMF by the focusing lens, which excites the PA waves within localized areas of the sample. To achieve compact configuration of the probe, the MMF was attached to the lensed SMF side by side using an epoxy glue. The diameter of the MMF was 660 μm, and the overall size of the probe was less than 900 μm.

### 2.4. Phantom Preparation

To evaluate the performance of the proposed system, we used a mixed solution of milk and water, containing a transparent fishing line (diameter of 300 μm) and a black polyethylene terephthalate (PET) fiber (BSHLR180, HellermannTyton, Incheon, Korea, diameter of 250 μm). The milk and water solution is generally used to model a scattering medium such as a tissue, because the main components of milk are fat and protein. For a 5% solution of milk and water, 5 mL of milk was diluted with 95 mL of water. After placing the fishing line and the black PET fiber at a petri dish in a cross configuration, the mixed solution was carefully poured onto them.

## 3. Experiment and Results

**Figure 3:**
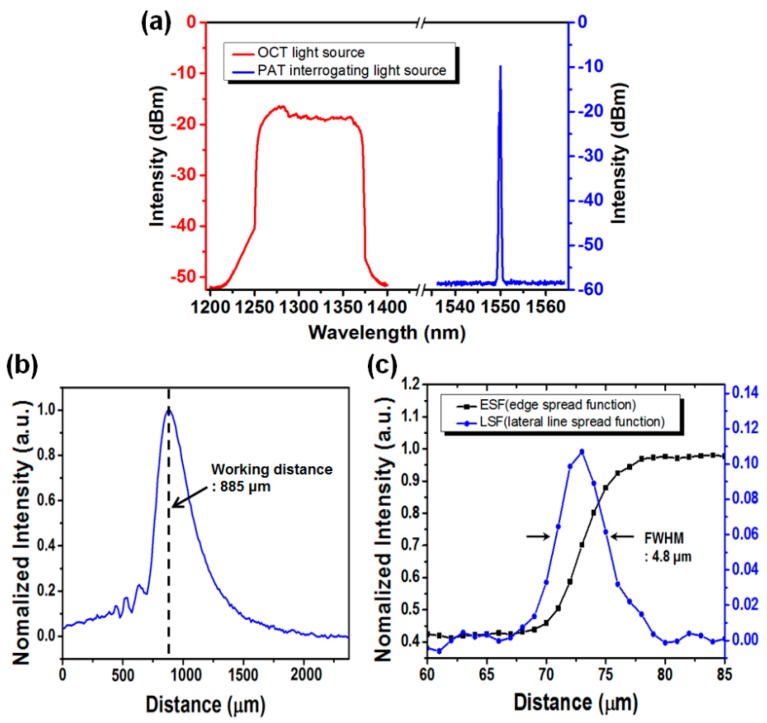
(**a**) The wavelength spectrum of the combined beam of OCT and PAT after passing wavelength division multiplexer (WDM); (**b**) The recoupling optical power of the light beam back reflected from a mirror moving away from the lens tip, plotted in terms of the mirror distance from the tip; (**c**) Edge spread function (ESF) obtained from a knife-edge mirror (square-symbol black line) and line spread function (LSF) obtained by taking the derivative of ESF (round-symbol blue line).

The commercial WDM, used for integration and separation of interrogating lights of PAT and OCT, was designed for 1550 nm and 1310 nm with a bandwidth of ±50 nm. In the experiment, the center wavelength of the PAT source was 1550 nm, and the one of the OCT source was centered at 1310 nm with a −20 dBm bandwidth of 110 nm. Figure 3a shows the transmission spectrum of the combined beam after passing through the WDM. We can see that, although the wavelength bandwidth of the OCT light source slightly exceeds the pass bandwidth of the WDM, the WDM still works reliably.

To evaluate the performance of the homemade-lensed fiber, we measured the working distance and the beam width at the focal plane of the fiber lens. The working distance, defined as the distance from the tip of the fiber to the focal point, was estimated from the plot of the optical coupling power. The optical power of the light back reflected from a mirror moving away from the lens tip was measured and plotted in terms of the mirror distance from the tip. As shown in Figure 3b, the working distance of the lensed fiber was measured as 885 μm. The beam width of the lensed fiber was estimated from the lateral line spread function (LSF) obtained by taking the spatial-derivative of the edge spread function (ESF) [24]. The ESF was measured at the working distance of the lensed fiber by moving a knife-edge mirror in a lateral direction, shown in Figure 3c with a square-symbol black line. By taking the derivative of ESF, the lateral LSF was obtained as shown with the round-symbol blue line in the figure. The full width at half maximum of the lateral LSF says that the beam width of the lensed fiber was approximately 4.8 μm. In an OCT system, in general, the lateral resolution of OCT is determined by the beam width of the sample beam at the focal point.

**Figure 4:**
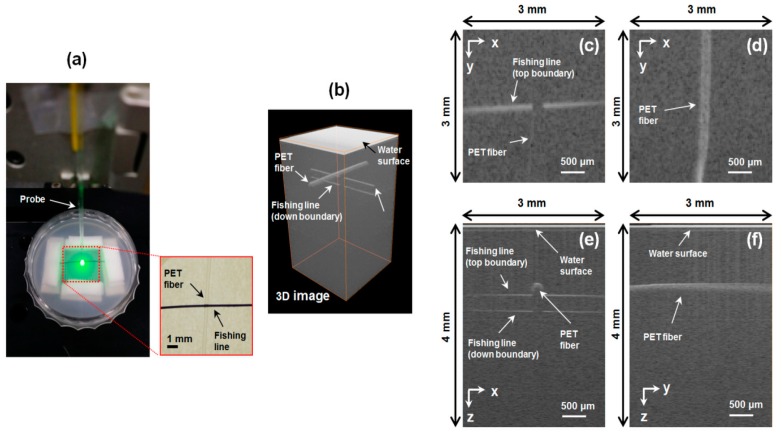
OCT measurements of the phantom. (**a**) Photograph of the sample arm and the phantom. The excitation area of the pulsed laser (wavelength of 532 nm) is seen with green color and the inset is a photograph of the phantom; (**b**) The obtained 3D OCT image of the phantom; (**c**) Cross-sectional image in the *xy* plane taken at the top boundary of the fishing line; (**d**) The same as (**c**) but taken at the black polyethylene terephthalate (PET) fiber; (**e**) Cross-sectional image in the *xz* plane taken at the cross point of the fishing line and PET fiber; (**f**) The same as (**e**) but taken along the orthogonal direction.

To investigate the feasibility of the proposed dual-modal PAT-OCT system having the common fiber probe, we have imaged the phantom made of a fishing line and a black PET fiber placed in the mixed solution of milk and water. Figure 4a shows a photograph of the fiber probe and the phantom used in the experiment. The inset of the figure shows that the fishing line and the black PET fiber are crossed in configuration. The radiant exposure of the pulsed Nd:YAG laser on the sample surface, for the PA excitation, was approximately 15.9 mJ/cm^2^; this is permitted by the American National Standards Institute regulations for laser safety of skin [25]. The fiber probe was positioned perpendicular to the phantom surface and scanned over an area of 3 mm × 3 mm in steps of 30 μm. The PA signals and the OCT signals were detected at the same position and at the same time. The PA signals were averaged 5 times per each detection point; however, the OCT signals were not averaged. Then, the PAT and OCT images were reconstructed by using the Fourier transform method, and some image processing was made [18,20].

At first, the reconstructed OCT images are shown with Figure 4. Figure 4b shows a three-dimensional (3D) OCT image volume-rendered by using a commercial software (Amira, FEI Co., Hillsboro, OR, USA), which matches well with the phantom structure in the inset of Figure 4a. The 3D OCT image clearly shows the water surface and the structure of both the fishing line and the black PET fiber, depicted with grayscale. For the detailed observation of OCT image, the cross-sectioned images in the *xy*, *xz*, and *yz* planes were reconstructed. The *xy* cross-sectional image Figure 4c taken at the top boundary of the fishing line and the *xz* image Figure 4e taken at the cross point show that the top and the bottom boundaries of the fishing line and the top boundary of the PET fiber are distinguishably visualized. However, the boundaries of the fishing line just beneath the PET fiber cannot be seen. It is due to the opaqueness of the black PET material. In Figure 4d, the cross-sectional *xy* image taken at the top of the PET fiber, and in 4f taken along the PET fiber, we can see only the upper part of the black PET fiber, which confirms the strong optical absorption at the black PET fiber.

**Figure 5:**
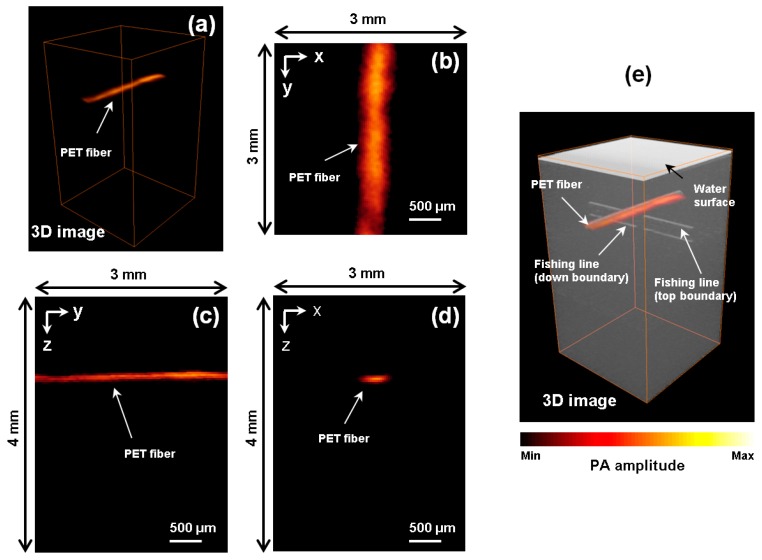
PAT measurement of the crossed fishing line and black PET fiber embedded in 5% solution of milk and water: (**a**) 3D PAT image of the phantom; (**b**–**d**) Cross-sectional images in the *xy* plane, *xz* plane, and *yz* plane at the center of the PET fiber; (**e**) Merged 3D PAT and OCT image. The 3D rendered images can be viewed online at Video 1.

Secondly, the reconstructed PA images acquired simultaneously with the OCT images are shown with Figure 5. Figure 5a shows the acquired 3D PA image of the phantom. In contrast with OCT, only the black PET fiber appears clearly. The transparent fishing line, which has low absorption at the wavelength of the pulsed laser, is invisible in the PA image. The cross-sectioned PA images in the *xy*, *yz*, and *xz* planes are reconstructed in Figure 5b–d, respectively. The PET fiber is clearly recognized in all directions and well matched with the actual location of the PET fiber in the phantom.

Finally, the volume-rendered PAT and OCT images are merged and presented together in Figure 5e. The PET fiber imaged by PAT shows its overall structure (top and bottom) but only the upper part of the PET fiber is shown in the OCT image. However, the fishing line could be identified only with OCT. PAT could not see the fishing line, mainly due to the low light absorption at the excitation wavelength (532 nm in our case). The configuration of the black PET fiber and the transparent fishing line in the merged PAT and OCT image are well matched with the actual structure of the phantom. The merged image confirms that the proposed system can provide the complementary information of the phantom—morphological (by OCT) and absorption (by PAT) information. The 3D merged image of PAT and OCT shown in Figure 5e is also available online (Video 1). The movie provides a further demonstration of the merged image.

## 4. Discussion

The proposed all-fiber-based PAT and OCT system can be used for the dual-modal endoscopy imaging owing to its compactness. However, for the realistic endoscopy, we still overcome several challenging problems.

Firstly, the imaging speed needs to be high enough to mitigate image artifacts caused by the sample movement and to image dynamic information. In PA imaging, the imaging speed depends on the repetition rate of the pulsed laser. To improve the PAT imaging speed, several studies have been reported with the usages of pulsed laser diodes (PLDs) [26,27,28]. The PLD has a high repetition rate (~kHz); however, the pulse energy is ~100 times lower than the pulse energy of Nd:YAG laser [28]. To solve this drawback of the PLD, arrays of PLDs have also been tried [26,27,28]. By using a fiber combiner, we are going to combine the beams from several PLDs into a single multimode fiber.

For an effective endoscopic application, an effective scanning method is required. In general, there are two types of scanning methods: forward-viewing and side-viewing [29,30]. Our proposed system has adopted the forward-viewing scanning method. The fiber probe was laterally scanned with a linear stage, which is relatively inappropriate for the realistic endoscopy imaging applications. For the realistic imaging, we consider the usage of a PZT scanner [31] or a magnetic force scanner [32] as the effective forward-viewing scanner.

The more compact and stable configuration of the common probe would be an in-line fiber probe instead of using two fibers; SMF for detection and MMF for PA excitation. For implementing the in-line fiber probe, we decided to replace the two fibers with a double cladding fiber (DCF) [33]. We can send the high-power PA excitation beam via the inner cladding of the DCF and the rather low-power detection beams of PAT and OCT via the core of the DCF. For the photoacoustic microscopy (PAM) case, the double cladding lensed fiber would be appropriate because the lateral resolution of PAM depends on the spot size of the excitation beam. However, for the PAT case, the PA excitation beam is generally illuminated without tight focusing. Therefore, we need to optimize the focal length of the common fiber lens if we try to use the in-line fiber probe.

The spot size of the beam for the PA detection was focused down to a few micrometers, much smaller than the measured lateral resolution. It means that the spot size of the PA detection beam is not the main factor in determining the lateral resolution of the current PAT system. Of course, at the surface of the sample, the lateral resolution is directly proportional to the spot size of the PA detection beam. Meanwhile, for the case of the optical resolution PAM, as was mentioned, the spot size and the depth of focus of the PA excitation beam would be important.

The feasibility of the proposed system was tested with a simple sample consisting of a few wires in a milk-water solution. For real cases, such as those of human tissue with a tumor, there are many light absorbers or PA emitters within a small volume. Therefore, we should consider the problem of crosstalk among so many signals from different sources for such real applications. However, it is not the problem of the optical scheme detection of the PA signal. If it exists, it is the problem of PAT itself. After first implementing a high-speed and high-resolution PAT system, we will then analyze the crosstalk issue of the PAT modality in the future.

Lastly, in the proposed dual-mode system, the lateral scanning interval of 30 μm was used due to the low imaging speed and the poor lateral resolution of PAT. Generally, the lateral resolution of OCT is high and determined mainly by a numerical aperture of the optics in the sample arm. However, the resolution of PAT is determined by various factors: element size of the ultrasonic transducer (in our case, spot size of the detection beam), bandwidth of the ultrasonic transducer, scanning length (field of view), scanning interval, and acoustic attenuation within the sample [12,28]. Therefore, increasing the imaging speed of the PAT can reduce the scanning interval so that the maximum resolution of OCT can be used.

## 5. Conclusions

We have successfully implemented an all-fiber-based dual-modal imaging system that integrated noncontact PAT and OCT subsystems. For the combined system, a common sample probe was made with a lensed single-mode fiber (SMF) and a large-core multimode fiber (MMF). The noncontact measurement of the PAT subsystem was achieved by utilizing the fiber interferometer, which measured the PA signal from the surface displacement of a sample. It was done through the lensed SMF along with the OCT measurement. For the efficient PA excitation, the large-core MMF was used. The PAT subsystem consisted of a fiber laser of 1550 nm for PA signal detection and a Q-switched Nd:YAG laser of 532 nm for PA excitation. It had the lateral resolution of 100 μm and axial resolution of 30 μm at an imaging depth of 2.5 mm. The fiber-based OCT subsystem used a swept-source laser with a center wavelength of 1310 nm, wavelength bandwidth of 110 nm, and a scan speed of 20 kHz. The fiber lens formed at the end of the SMF (through a piece of 600 μm long and 180 μm diameter CSF) gave a working distance of 885 μm and a beam width of 4.8 μm at the focal plane. By attaching the lensed SMF with the 660-μm-thick MMF side by side, the common fiber probe could be implemented with a diameter less than 900 μm. The fiber-optics configuration of both systems is absolutely valuable for integrating both systems. By using the common fiber probe, the combined system could be highly miniaturized.

To evaluate the proposed system, we obtained and presented PAT and OCT images, and the merged one, of a phantom at the same position and at the same time. The phantom consisted of a fishing line and a black PET fiber embedded in a tissue mimicking solution. The merged 3D image of PAT and OCT showed that the OCT could see both the fishing line and the PET fiber, but the PAT saw only the PET fiber. Thus, we can conclude that the proposed system can provide the complementary information of the sample related to optical absorption and scattering. Considering the advantages of fiber-optic configuration and noncontact PA measurement, we expect that the proposed all-fiber PAT–OCT system can be utilized as a medical diagnostic tool in fields requiring noncontact, minimally invasive and endoscopic imaging.

## Supplementary Materials

The following are available online at http://www.mdpi.com/1424-8220/16/5/734/s1.

## Abbreviations

The following abbreviations are used in this manuscript:

PAT	photoacoustic tomography
OCT	optical coherence tomography
PET	polyethylene terephthalate
SMF	single-mode fiber
CSF	coreless silica fiber
MMF	multimode fiber
AOM	acousto-optic modulator
WDM	wavelength division multiplexer
LSF	line spread function
ESF	edge spread function
